# Antibacterial Dental Adhesive Containing Cetylpyridinium Chloride Montmorillonite

**DOI:** 10.3390/ma17174368

**Published:** 2024-09-04

**Authors:** Yohei Okazaki, Kiichi Nakamori, Chenmin Yao, Mohammed H. Ahmed, Benjamin Mercelis, Noriyuki Nagaoka, Yukinori Maruo, Yasuhiro Yoshida, Yasuhiko Abe, Bart Van Meerbeek, Kumiko Yoshihara

**Affiliations:** 1 Department of Oral Health Sciences, BIOMAT, KU Leuven, 3000 Leuven, Belgium; okazaki-yoh@hiroshima-u.ac.jp (Y.O.); chenmin.yao@kuleuven.be (C.Y.); m.ahmed@dent.tanta.edu.eg (M.H.A.); benjamin.mercelis@kuleuven.be (B.M.); 2 Department of Advanced Prosthodontics, Graduate School of Biomedical and Health Sciences, Hiroshima University, Hiroshima 734-8553, Japan; nkiichi@hiroshima-u.ac.jp (K.N.); abey@hiroshima-u.ac.jp (Y.A.); 3 Advanced Research Center for Oral and Craniofacial Science, Okayama University Dental School, Okayama 700-8558, Japan; nagaoka@okayama-u.ac.jp; 4 Department of Prosthodontics, Okayama University, Okayama 700-8558, Japan; ykmar@okayama-u.ac.jp; 5 Department of Biomaterials and Bioengineering, Faculty of Dental Medicine, Hokkaido University, Sapporo 060-8586, Japan; yasuhiro@den.hokudai.ac.jp; 6 Health and Medical Research Institute, National Institute of Advanced Industrial Science and Technology (AIST), Takamatsu 761-0395, Japan; 7 Department of Pathology & Experimental Medicine, Graduate School of Medicine, Dentistry and Pharmaceutical Sciences, Okayama University, Okayama 700-8558, Japan

**Keywords:** dental adhesive, antibacterial agent, dentin, degree of conversion, micro tensile bond strength, scanning microscopy

## Abstract

Oral bacteria cause tooth caries and periodontal disease. Much research is being conducted to prevent both major oral diseases by rendering dental materials’ antimicrobial potential. However, such antimicrobial materials are regarded as ‘combination’ products and face high hurdles for regulatory approval. We loaded inorganic montmorillonite with the antimicrobial agent cetylpyridinium chloride, referred to below as ‘CPC-Mont’. CPC-Mont particles in a 1, 3 and 5 wt% concentration were added to the considered gold-standard self-etch adhesive Clearfil SE Bond 2 (‘CSE2’; Kuraray Noritake) to render its antibacterial potential (CSE2 without CPC-Mont served as control). Besides measuring (immediate) bonding effectiveness and (aged) bond durability to dentin, the antibacterial activity against *S. mutans* and the polymerization-conversion rate was assessed. Immediate and aged bond strength was not affected by 1 and 3 wt% CPC-Mont addition, while 5 wt% CPC-Mont significantly lowered bond strength and bond durability. The higher the concentration of the antimicrobial material added, the stronger the antimicrobial activity. Polymerization conversion was not affected by the CPC-Mont addition in any of the three concentrations. Hence, adding 3 wt% CPC-Mont to the two-step self-etch adhesive rendered additional antimicrobial potential on top of its primary bonding function.

## 1. Introduction

Dental caries remains a serious global health problem, affecting a large portion of the population [1,2,3]. Of particular concern is caries recurrence around tooth restorations, which, despite advances in dental care, still has a high incidence [1,2,3,4]. Repeated tooth re-treatment because of caries recurrence will expedite tooth loss [5,6]. To reduce caries recurrence, researchers have developed dental adhesives and restoratives that incorporate antimicrobial agents [7,8]. However, enhanced release of antimicrobials carries the risk of increased toxicity [7,8,9]. Furthermore, because antimicrobial agents are considered as pharmaceutical agents, they require rigorous evaluation and approval by regulatory agencies such as the U.S. Food and Drug Administration (FDA), the European Medicines Agency (EMA) and the Pharmaceuticals and Medical Devices Agency (PMDA) in Japan, complicating their production and marketing [10].

Despite numerous studies aimed at combating tooth caries, only a few products with cariogenic potential have successfully been marketed [11]. One such product is Clearfil SE Protect (Kuraray Noritake Dental, Tokyo, Japan), a dental adhesive designed to reduce caries recurrence in adhesive restorations [11,12]. While an in vitro antibacterial effect has repeatedly been proven, the ultimate clinical efficacy of Clearfil SE Protect (Kuraray Noritake Dental) in reducing caries recurrence has not been demonstrated [13]. This highlights an ongoing challenge in the development of antimicrobial dental materials that are clinically effective. Thus, there exists a need for innovative biosafe solutions to reduce/prevent secondary caries [14,15,16,17].

To meet this challenge, we have developed novel antimicrobial technology based on montmorillonite loaded with cetylpyridinium chloride, as referred to here as ‘CPC-Mont’ [18,19,20]. This CPC-Mont technology has already been included into a commercial tissue conditioner for short-term use (Tissue Conditioner CPC, Morita, Osaka, Japan) [19]. This tissue conditioner was successfully demonstrated in vitro to possess antimicrobial efficacy against *Candida albicans*, *Streptococcus mutans* and *Staphylococcus aureus*. Having been approved by the PMDA as a dental device with additional pharmaceutical efficacy, the antimicrobial properties of Tissue Conditioner CPC (Morita) have officially been claimed and mentioned in the package insert [19,21].

Based on the successful use of CPC-Mont in a tissue conditioner, we now aimed to incorporate CPC-Mont technology into the considered gold-standard two-step self-etch (SE) adhesive Clearfil SE Bond 2 (C-SE2;Kuraray Noritake Dental), to render its antibacterial properties. Three experimentally formulated dental adhesives containing 1, 3 and 5 wt% CPC-Mont were subjected to a series of in vitro experiments, among which were the measurement of bond strength to dentin and, antibacterial activity against the most common cariogenic micro-organism *S. mutans*, and polymerization conversion. The hypotheses tested were that the addition of CPC-Mont to the adhesive (1) did not affect bond strength to dentin, as its primary function, (2) did render the adhesive antibacterial efficacy, and (3) did not reduce polymerization conversion.

## 2. Materials and Methods

### 2.1. Antibacterial Agent Preparation

CPC-Mont (Medical Crafton, Okayama, Japan) was prepared as described in detail before [12,13,14]. In brief, 2 g of Na-Mont (Kunipia F, Kunimine Industries, Tokyo, Japan) was dispersed in deionized water (200 cm^3^) and stirred for 1 h. A pre-dissolved stoichiometric amount of CPC corresponding to four times the cation exchange capacity of Na-Mont was slowly added to the dispersion. Then, the dispersion was stirred for 1 d at room temperature. Solid products were separated by centrifugation, washed with deionized water and dried at 60 °C for 1 d. Finally, the sample was ground to powder. CPC-Mont particles varying in size between 5 and 25 µm were added to the adhesive resin (‘Bond’) of C-SE2 in a 1, 3 and 5 wt% CPC-Mont concentration. C-SE2 without CPC-Mont served as control. C-SE2’s primer was used in all experimental groups.

### 2.2. Antibacterial Efficacy

**Figure 1:**
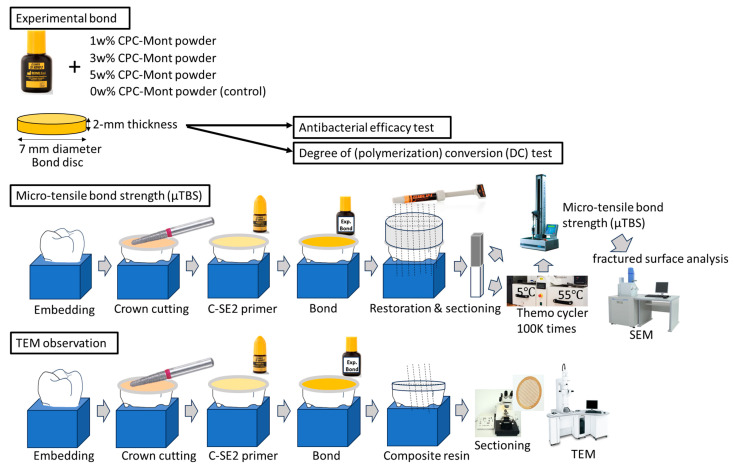
Schematic diagram for this study.

C-SE2 Bond discs were made using Teflon molds with a 7 mm diameter and 2 mm thickness (Figure 1). To avoid polymerization inhibition by oxygen, a glass slide was positioned on top of the mold, upon which the C-SE2 Bond discs were light-cured at the center through the glass for 60 s using the polywave light-emitting diode (LED) light-curing unit Bluephase 20i (Ivoclar, Schaan, Liechtenstein) with an output of 1200 mW/cm^2^ when used in ‘high mode’, as determined and confirmed using a Marc Resin Calibrator (BlueLight Analytics, Halifax, NS, Canada). After removing the discs from the mold, the bottom of the discs was additionally light-cured for 60 s. Next, the disc top and bottom were UV-irradiated for 3 h for sterilization.

Overnight cultures of *Streptococcus mutans* (ATCC 25175) were centrifuged and resuspended in fresh Brain Heart Infusion broth (BHI, Becton Dickinson and Company, Franklin Lakes, NJ, USA). The concentration of the bacterial suspension was adjusted spectrophotometrically (600 nm) (BioSpectrometer Basic, Eppendorf, Hamburg, Germany) to 2 × 10^7^ CFU/mL. Five adhesive discs were placed in 48 well plates (one specimen with 300 µL BHI per well). A total of 2 mL *S. mutans* suspension was added into the wells. As positive and negative control, three wells did not receive an adhesive disk and another three wells did not receive the bacterial suspension. Specimens with *S. mutans* cultures and with sterile BHI broth were incubated (37 °C, 5% CO_2_) under aerobic conditions. The 600 nm absorbance was measured at 24 h and 37 °C using a microplate reader (POLARstar Omega, BMG Labtech, Ortenberg, Germany).

The 24 h bacterial growth data were statistically compared with one-way analysis of variance (ANOVA) and Tukey’s HSD test at the significance level of α = 0.05, using IBM SPSS Statistics (version 25; IBM).

### 2.3. Degree of (Polymerization) Conversion (DC)

The specimens, prepared in the same way as for measuring antibacterial efficacy, were transferred to a micro-Raman spectrometer (Senterra, BrukerOptik, Ettlingen, Germany) (Figure 1). Micro-Raman spectra of each specimen were collected at 24 h after light-curing, using the following settings: 785 nm Ar-ion laser, 100 mW power, 1200 grooves/mm diffraction grating, 50 µm pinhole aperture, and a 100× objective. The spectrum-integration time was 20 s, with the recorded spectra averaged over two successive measurements. Each specimen was measured at least twice. All specimens were kept dry and in the dark at 37 °C at all times.

DC was calculated from the height of the aliphatic C=C peak at 1638 cm^−1^ and the reference peak, according to the following formula:

DC% = (1 − (C_aliphatic_/C_reference_)/(U_aliphatic_/U_reference_)) × 100 (%)

with C_aliphatic_ being the absorption peak at 1638 cm^−1^ of the cured specimen, Creference the reference peak of the cured specimen, U_aliphatic_ the absorption peak at 1638 cm^−1^ of the uncured specimen, and U_reference_ the reference peak of the uncured specimen. The fraction of remaining C=C double bonds for each spectrum was determined after baseline correction, comparing the maximum heights of the aliphatic and reference peaks using OPUS 7.0 software (BrukerOptik).

The DC data were statistically compared with one-way analysis of variance (ANOVA) and Tukey’s HSD test at the significance level of α = 0.05, using IBM SPSS Statistics (version 25; IBM Corp., Armonk, NY, USA).

### 2.4. Micro-Tensile Bond Strength (µTBS)

The schematic illustration of micro-tensile bond strength (μTBS) is given in Figure 1. Tooth crowns, 40 in total, were cut 4 mm below the cusp tops, ending within mid-coronal dentin. In this way, the effect of regional variability on µTBS was kept minimal. All surfaces were next prepared using the MicroSpecimen Former (University of Iowa, Iowa, IA, USA), equipped with a high-speed medium-grit (107 µm) diamond bur, hereby producing a standard dentin smear layer. On the flat dentin surfaces, build-ups in composite resin (Clearfil AP-X, Kuraray Noritake) were next made, in incremental layers of 2 mm. The adhesive and composite were applied, following strictly the manufacturer’s instructions. The adhesive and each composite increment were light-cured using a light-emitting diode (LED) light-curing unit (Bluephase 20i, Ivoclar, Schaan, Liechtenstein) with an output of 1200 mW/cm^2^ when used in ‘high mode’, as determined and confirmed regularly during the experiment, using a Marc Resin Calibrator (BlueLight Analytics, Halifax, NS, Canada). Output was measured each time before and after preparation of each experimental group-specimen set. Once prepared, the bonded specimens were kept for 1 h at 100% humidity prior to having been immersed for 1 week in distilled water at 37 °C. After 1-week water storage, all specimens will be sectioned, perpendicular to the interface, using a water-cooled diamond saw (Accutom-50, Struers, Ballerup, Denmark) to obtain rectangular sticks (at least 4 micro(µ)-specimens per tooth, half with the following dimensions: 1 × 1 mm wide; 8–9 mm long). Half of the µ-specimens were tested immediately, to determine µTBSimmediate, while the other half µ-specimen set was stored for long-term 100 k thermocycling between 5 °C and 55 °C, prior to being subjected to µTBS testing to determine µTBSaged.

For the actual µTBS test, the specimens were fixed to a BIOMAT jig using cyanoacrylate-based glue (Model Repair II Blue, J. Morita Corp., Osaka, Japan) and stressed at a crosshead speed of 1 mm/min until failure in an LRX testing device (LRX, Lloyd, Hampshire, UK) using a load cell of 100 N. When specimens failed before actual testing, they were recorded as pre-testing failures (ptf’s). Following conventional SEM-specimen processing, including fixation (2.5% glutaraldehyde in 0.1 M sodium cacodylate buffer), gradual dehydration in ethanol (25%, 50%, 75%, 95%, 100), and drying with hexamethydisilazane (HMDS), fractured specimens representing each experimental group (and all ptfs) were selected and gold-sputter coated (JFC-1300, Jeol, Tokyo, Japan) before being examined using scanning electron microscopy (SEM, JSM-6610LV, Jeol).

The µTBS data were statistically analyzed with two-way ANOVA and Tukey’s HSD test at α = 0.05, using IBM SPSS Statistics (version 25; IBM).

### 2.5. TEM Interfacial Adhesive–Dentin Characterization

The schematic illustration of TEM sample preparation is given in Figure 1. Flat bur-cut dentin surfaces from two teeth were prepared as detailed above. After priming with C-SE2 primer for 10 s, followed by gently air-blowing (manufacturer’s instructions), the experimental 5 wt% CPC-Mont containing C-SE2 ‘Bond’ was applied for 20 s and gently air-blown prior to 10 s light curing. A 1 mm-thick layer of Clearfil Protect Liner Bond F (Kuraray Noritake) was applied on top of the adhesive layer, and it was light-cured again (for 20 s). The teeth were next stored in distilled water at 37 °C for 24 h before being cut using the diamond saw (Accutom-50, Struers) into 0.6-to-0.8 mm-thick slabs. The slabs were immersed in fixation solution of 2.5% glutaraldehyde in 0.1 M sodium cacodylate for 12 h, rinsed in 0.1 M sodium cacodylate buffer for 1 min with 3 changes, dehydrated in ascending grades of ethanol (25%, 50%, 75%, 95% and 100%) 2 times each and 10 min per treatment, immersed in 99% propylene oxide 3 times with 10 min per treatment, and finally embedded in epoxy embedding medium (Sigma-Aldrich, Saint Louis, MO, USA). Ultrathin sections of 70–90 nm were prepared using an ultramicrotome (Ultracut UCT, Leica, Vienna, Austria), equipped with a 45-degree diamond knife (Diatome, Nidau, Switzerland), prior to being examined with TEM (JEM-1400 Flash, Jeol).

## 3. Results

### 3.1. Antibacterial Efficacy

**Figure 2:**
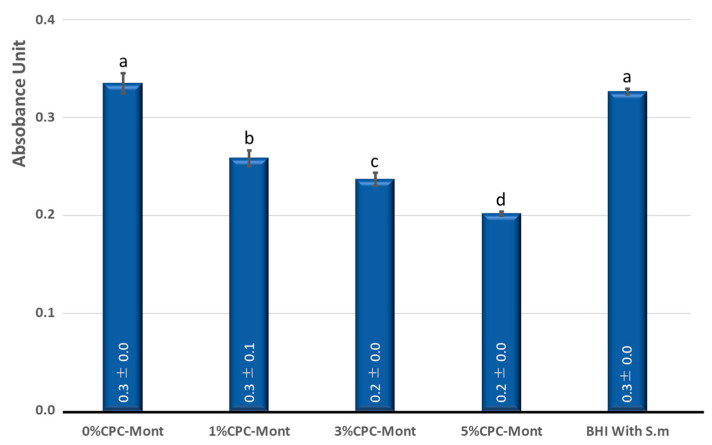
*Streptococcus mutans* (S.m.) growth on adhesive disks prepared with Clearfil SE Bond 2 (‘C-SE2’; Kuraray Noritake) ‘Bond’ containing 0 wt% (C-SE2 Bond without CPC-Mont as control), 1 wt%, 3 wt% and 5 wt% CPC-Mont, versus that of the test control (medium with *Streptococcus mutans* (BHI With S.m)); this was measured using optical density (OD in Absorbance Units) after 24 h incubation. The same letter above the bars indicates absence of significant difference (*p* > 0.05).

Testing the antimicrobial efficacy of the experimental C-SE2 ‘Bond’ disks, the *S. mutans* growth at 24 h revealed the significantly highest growth for the commercially available Clearfil SE Bond 2 (Kuraray Noritake) control without (0 wt%) CPC-Mont (Figure 2). The same turbidity (OD) was measured after 24 h as when *S. mutans* was allowed to grow on broth medium, indicating full absence of antimicrobial activity. Adding CPC-Mont to the experimental C-SE2 ‘Bond’ formulations significantly reduced *S. mutans* growth in a concentration-dependent pattern: the higher the added CPC-Mont concentration, the more the *S. mutans* growth was reduced.

### 3.2. Degree of Conversion (DC)

**Figure 3:**
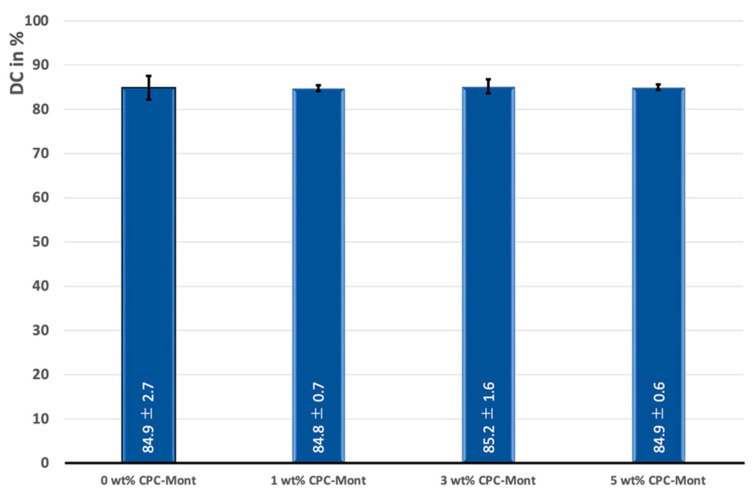
Degree of conversion (DC in %) of the experimental C-SE2 ‘Bond’ adhesive-resin formulations containing 0 wt% (control), 1 wt%, 3 wt% and 5 wt% CPC-Mont, as measured by micro-Raman spectroscopy. No significant differences in DC were recorded (*p* > 0.05).

No significant difference in 24 h DC was recorded for the experimental C-SE2 ‘Bond’ formulations containing CPC-Mont, as compared to the commercial version without CPC-Mont (Figure 3).

### 3.3. Micro-Tensile Bond Strength (µTBS)

**Figure 4:**
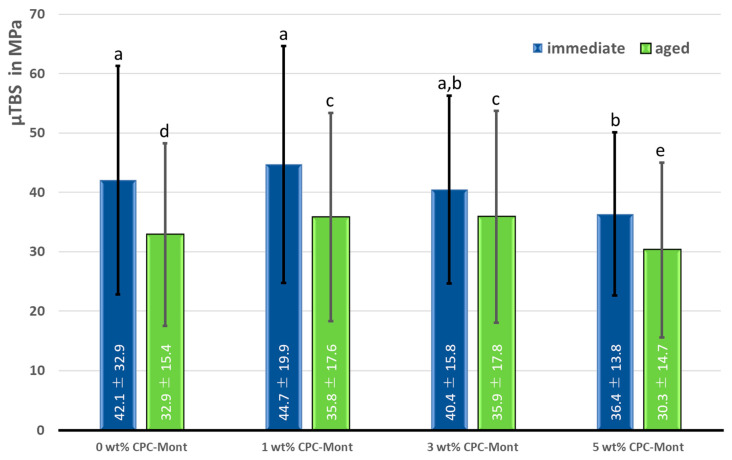
Micro-tensile bond strength (µTBS in MPa) of the experimental two-step self-etch C-SE2 adhesive formulations containing 0 wt% (commercial C-SE2 without CPC-Mont), 1 wt%, 3 wt% and 5 wt% CPC-Mont in relation to dentin after water storage for 1 week (µTBSimmediate) and 100 k thermocycles (µTBSaged). The same letter above the bars indicates absence of significant difference (*p* > 0.05).

Micro-tensile bond strength (µTBS) test results for 1-week water storage after sample preparation and 100 k after thermal cycle loading are shown in Figure 4.

Regarding µTBSimmediate, no significant difference was recorded between the 1 wt% and 3 wt% CPC-Mont-containing C-SE2 formulations and the commercial C-SE2 control (0 wt% CPC-Mont). However, adding 5 wt% CPC-Mont resulted in significantly lower µTBSimmediate, though not significantly different from that measured for the 3 wt% CPC-Mont formulation.

A significantly higher µTBSaged was recorded for the 1 wt% and 3 wt% CPC-Mont-containing C-SE2 formulations as compared to the commercial C-SE2 control (0 wt% CPC-Mont). A significantly lower µTBSaged was recorded when 5 wt% CPC-Mont was added, as compared to all other experimental and control groups.

### 3.4. SEM Fracture-Mode Analysis

**Figure 5:**
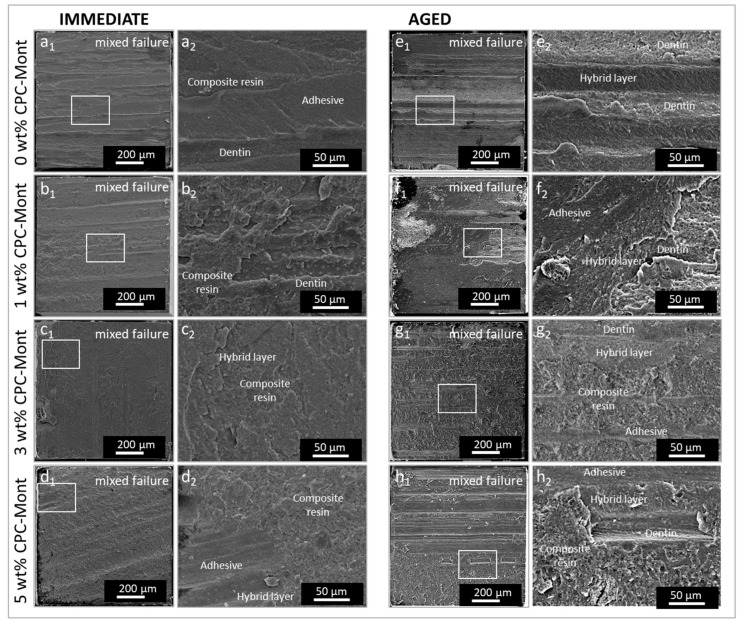
Representative SEM photomicrographs of fractured µTBS micro-specimen surfaces of the experimental two-step self-etch C-SE2 adhesive formulations containing 0 wt% (commercial C-SE2 without CPC-Mont), 1 wt%, 3 wt% and 5 wt% CPC-Mont, as bonded to dentin, of which the µTBS was measured ‘immediately’ after 24 h water storage (**a_1_**–**d_2_**), and upon aging after long-term 100 k thermocycles. (**e_1_**–**h_2_**). Each white rectangle is magnified in the right adjacent photomicrograph.

The fracture surface of all adhesion tests was observed. Among them, SEM images of representative fracture surfaces of each group are shown in Figure 5. SEM fracture-mode analysis revealed a greater effect of ageing than that caused by difference in experimental adhesive formulation. The dentin side of a representative ‘mixed failure’ of 0 wt% CPC-Mont Adhesive in is shown in a1, with a high magnification shown in a2. In a2, adhesive, dentin is clearly visible and composite resin filler can also be observed. Dentin side of a representative ‘mixed failure’ of 1 wt% CPC-Mont Adhesive in b1, with a high magnification shown in b2. The dentin side of a representative ‘mixed failure’ of 1 wt% CPC-Mont Adhesive in is shown in b1, and the composite resin and dentin can also be observed, with a high magnification shown in b2. The fracture surfaces of 3 wt% CPC-Mont Adhesive and 5 wt% CPC-Mont Adhesive also show a mixture fracture (c1 and d1, respectively). In the respective high-magnification sections, hybrid layers, composite resin and adhesive are observed (c2 and d2). Most ‘immediate’ fractures revealed a mixed failure pattern, including not only adhesive failure at the interface, but also cohesive failure within the adhesive or composite resin. In all experimental groups, traces of bur cutting were observed, suggesting that the fracture occurred close to the actual interface with dentin. Although the aging specimen also revealed mixture failure, the fracture surface was not similar to the ’immediate’ fracture. The dentin side of a representative ‘mixed failure’ of 0 wt% CPC-Mont Adhesive in e1, with a high magnification, is shown in e2. In e2, dentin is clearly visible, with dentin tubes, and a hybrid layer can also be observed. Compared to the fracture surface of a2, that of e2 revealed more within the hybrid layer. The dentin side of a representative ‘mixed failure’ of 1 wt% CPC-Mont Adhesive in f1, with high magnification observation in f2, reveals a more complicate surface, with adhesive, hybrid layer and dentin. The 1 wt% CPC-Mont Adhesive with ‘aging’ revealed a fracture within the hybrid layer, compared to the ‘immediate’ 1 wt% CPC-Mont Adhesive. The fracture surfaces of 3 wt% CPC-Mont Adhesive and 5 wt% CPC-Mont Adhesive with aging also show a mixture fracture (g1 and h1, respectively). High-magnification observations reveal the presence of the hybrid layer, dentin, and adhesive in both g2 and h2. A mixed failure mode was also observed upon aging, with the interface failing more within the hybrid layer.

### 3.5. TEM Interfacial Adhesive–Dentin Characterization

**Figure 6:**
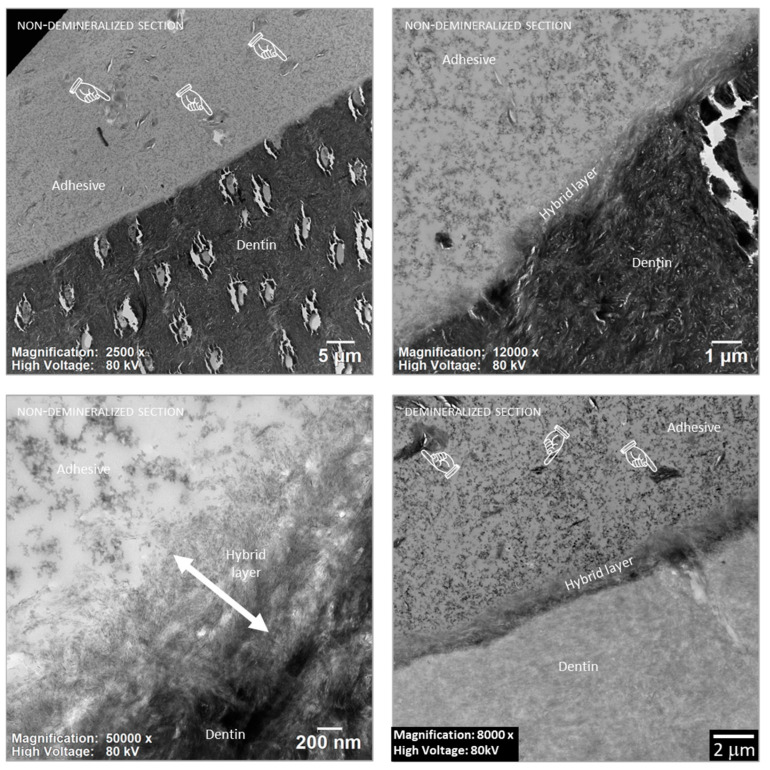
Cross-sectional transmission electron microscopy (TEM) image of the adhesive–dentin interface produced by the experimental C-SE2 adhesive formulation with 5 wt% CPC-Mont added to the adhesive-resin (C-SE2 ‘Bond’). The hand pointers show the relatively homogenous dispersion of CPC-Mont within the adhesive.

To assess the dispersion of CPC-Mont within the adhesive material, we employed TEM to examine the adhesive interface of the sample containing the highest concentration of 5 wt% CPC-Mont (Figure 6). CPC-Mont particles were observed to have been distributed relatively uniformly within the adhesive-resin layer. TEM did not reveal differences in interfacial ultra-structure produced at the dentin by the experimental C-SE2 adhesive formulation containing 5 wt% CPC-Mont.

## 4. Discussion

In this study, we tested a considered gold-standard self-etch adhesive to which CPC-Mont antimicrobial filler technology was added. CPC-Mont technology has been introduced before, and its antibacterial effectiveness has already been shown when it was added to a denture tissue conditioner [19], luting composite [20] and one-step adhesive [18]. When 2% CPC-Mont was added to the tissue conditioner, antibacterial activity persisted for 1 week against C. albicans; against S. aureus, bacterial growth was inhibited for up to 3 weeks [19]. The clay montmorillonite has a layered and electrically charged structure, by which electrically charged molecules like CPC, being antibacterial, can be uploaded within the layered structure. As a carrier, montmorillonite can release CPC, rendering resin-based dental materials antibacterial, and thus having anticariogenic potential [18]. When CPC-Mont was added to cement, the higher CPC-Mont content showed longer-lasting antimicrobial activity: cements containing 5 or 7.5 w% CPC-Mont inhibited biofilm formation for 30 days [20]. When CPC was added to one-step adhesive at concentrations of 1% or 3% alone, its antimicrobial activity was not significantly suppressed. However, when CPC-Mont was added at the same concentrations, it demonstrated antimicrobial activity [18].

In this study, the antibacterial effectiveness of experimental CPC-Mont adhesive formulations against the cariogenic pathogen *Streptococcus mutans* was recorded when CPC-Mont was added in a 1-to-5 wt% concentration. While the highest antibacterial effect was recorded when 5 wt% CPC-Mont was added to the adhesive, this experimental adhesive formulation presented with a lower bonding effectiveness and bond durability.

TEM revealed a relatively uniform dispersion of about 5 µm-sized CPC-Mont filler within the adhesive–resin layer immediately adjacent to the hybrid layer. This CPC-Mont filler is the same as that used in the antibacterial tissue conditioner (soft denture liner) which was marketed in Japan as ‘Tissue Conditioner CPC’ by Morita (Osaka, Japan). This denture liner is primarily composed of methacrylate-based polymers and fatty acid-based plasticizers. A tissue conditioner has a relatively sparse structure, allowing CPC to leach from inside the tissue conditioner. While a tissue conditioner has a thickness of a few millimeters, the film thickness of dental adhesives can vary considerably, but in a range of only micrometers. CPC-Mont is sufficiently small, and was added to the adhesive resin in a relatively low amount (in this study up to 5 wt%) so that it did not alter the adhesive’s film thickness.

Evaluation of antibacterial effectiveness revealed a dose dependency, with the more CPC-Mont added, the higher the antibacterial effectiveness recorded. In this study, aimed to develop dental adhesive technology to prevent caries recurrence around adhesive restorations, *S. mutans* was selected as the cariogenic pathogen, as being commonly used to test antibacterial effectiveness of dental materials and being the microorganism mostly associated with tooth caries. This study revealed findings regarding antimicrobial activity of CPC-Mont added to a two-step self-etch dental adhesive that were similar to those observed in previous studies when CPC-Mont was added to a one-step adhesive [18].

When CPC-Mont was added to the one-step adhesive [18], efficient antimicrobial activity was recorded, regardless of CPC-Mont concentration. In this study, antimicrobial activity was strongly dependent on the amount of CPC-Mont filler added to the adhesive resin of the two-step self-etch adhesive. Besides a two- versus one-step adhesive configuration, both bonding materials differ strongly in composition, meaning that the difference in antibacterial potential recorded must most likely be attributed to these compositional differences. In the previous study [18], the one-step adhesive investigated contained hydrophilic monomers dissolved in solvent, among which was water. Although adhesives are commonly air-blown upon their application, all the solvent cannot be expected to be removed from the adhesive interface, especially in the case of water [22]. Hence, the one-step adhesive was more hydrophilic than the two-step adhesive investigated [23,24]. Indeed, the adhesive resin of C-SE2, besides containing ethanol as solvent, also contains the much-more-hydrophobic monomer, probably also in higher concentrations (not released by the manufacturer) [25]. Solely the fact of being of a more hydrophobic nature may already explain the lower antimicrobial effectiveness recorded in this study, as this must significantly have influenced the elution behavior of CPC. Namba et al. (2009) reported before that when CPC only was added to adhesive resin, also as part of a two-step adhesive, elution of CPC was hardly detected, meaning that the antibacterial effect was limited to a surface-contact effect [26]. In the Namba et al. (2009) study, CPC was added, and thus it is ‘as if it is’ embedded within the adhesive resin [26], while in this study CPC was loaded into montmorillonite clay to gradually release CPC to induce longer-term antimicrobial activity. CPC is supported on montmorillonite in CPC-Mont; CPC accounts for about half of the weight of CPC-Mont. In the Namba et al. study, CPC was added in a 1 and 3 wt% concentration, whereas in this study CPC-Mont was added in 1, 3, and 5 wt%. Comparing the amount of CPC in CPC-Mont in the present study with the 1, 3 wt% CPC in Namba’s paper, the amount of CPC in the present study is slightly lower. In this study, the antimicrobial test is investigated in Brain Heart Infusion (BHI) medium. CPC-Mont exhibits antimicrobial activity, due to the sustained release of CPC loaded on montmorillonite. In the oral cavity, the sustained release of CPC can be affected by different pH levels and saliva. However, CPC is already used in mouthwashes and has also been found to be clinically effective in inhibiting plaque adhesion and gingival inflammation [27]. Tissue conditioners with CPC-Mont (Tissue Conditioner CPC) have been used in in vitro antimicrobial studies using culture media, and clinical plaque inhibition has also been reported [19,28,29]. Although the antimicrobial properties of this antimicrobial-containing adhesive material ultimately require clinical evaluation, it is expected to be effective in the actual oral cavity, given the antimicrobial properties of the CPC itself and the results of the preceding products. However, the amount of CPC-Mont to be added should not be determined based only on the antimicrobial properties targeted, but also in light of retaining sufficient mechanical strength, among which properties bond strength is also a primary function of a dental adhesive.

The polymerization rate of a resin-based material directly (co-)determines material strength and durability. Hence, the polymerization rate (DC) should always be as high as possible. This study revealed that adding CPC-Mont up to a concentration of 5 wt% did not affect the polymerization rate of the adhesive investigated, which did not even appear lower than that of the commercial adhesive to which no CPC-Mont was added. The addition of CPC-Mont to the one-step adhesive in the previous study [18] also did not affect the DC. CPC-Mont is small in size, up to 5 µm, as observed by TEM in this study. This CPC-Mont filler size falls within the range of a common composite-resin filler, and is not thought to affect light curing.

Bond strength and durability were measured using a micro-tensile bond-strength test [29,30,31]. Bond durability was measured upon long-term 100 k thermocycling. Obviously, differences in bonding performance will more easily be recorded when the micro-specimens are challenged more severely or aged longer. While bonding effectiveness measured ‘immediately’ after 1 week of water storage was not statistically different for the adhesive formulations containing 1 and 3 wt% CPC-Mont as compared to that of the commercial adhesive (control) to which no CPC-Mont was added, a significantly lower µTBSimmediate was recorded for the 5-wt% CPC-Mont adhesive formulation. Upon long-term thermocycling aging, bond strength decreased for all adhesive formulations. Again, no significant difference in µTBSaged was recorded among the 1 and 3 wt% CPC-Mont adhesive formulations, even outperforming the control not containing CPC-Mont. As recorded for µTBSimmediate, adding 5 wt% CPC-Mont significantly reduced the µTBSaged.

Although the polymerization conversion of C-SE2’s adhesive resin was not decreased by adding CPC-Mont, the bonding material still absorbs water, and thus may undergo hydrolytic degradation [32]. In this study, adhesive–resin disks were subjected to long-term thermocycling and thus exposed to water. In particular, montmorillonite may be susceptible to degradation because of its high water sorption. Hence, the lower µTBSaged recorded for the 5 wt% CPC-Mont adhesive formulation may, at least in part, also be ascribed to the higher water sorption of CPC-Mont. SEM fracture analysis appeared to confirm this reasoning, as most specimens failed in a mixed failure mode, without much difference observed among the experimental adhesive formulations. The ‘immediate’ µTBS micro-specimens revealed a mixed failure pattern including more fractures within composite or adhesive resin, while the ‘aged’ micro-specimens revealed a mixed failure pattern including more fractures within the hybrid layer. The failure-mode pattern changed upon thermocycling aging, which may be due to temperature- and water-sorption-induced effects [32,33,34]. Methacrylate resins have been reported to have different strength, durability and cytotoxicity, depending on their crosslinking agents and monomer formulations [35]. By modifying the composition of methacrylate resins, it may be possible to find compositions with even higher strength and durability, sufficient antimicrobial activity, and lower toxicity than C-SE2.

## 5. Conclusions

In conclusion, this study confirmed that adding CPC-Mont rendered the considered gold-standard two-step self-etch adhesive antimicrobial properties. Higher CPC-Mont loading increased the antimicrobial activity but also affected bonding effectiveness, while not affecting polymerization conversion. Adding 3 wt% CPC-Mont to the two-step self-etch adhesive, the C-SE2 investigated was most optimal, having resulted in the highest antibacterial potential without reducing its bonding effectiveness. 

## Data Availability

The original contributions presented in the study are included in the article, further inquiries can be directed to the corresponding author.
